# The relationship between endogenous oxytocin and vasopressin levels and the Prader-Willi syndrome behaviour phenotype

**DOI:** 10.3389/fendo.2023.1183525

**Published:** 2023-05-29

**Authors:** Lauren J. Rice, Josephine Agu, C. Sue Carter, James C. Harris, Hans P. Nazarloo, Habiba Naanai, Stewart L. Einfeld

**Affiliations:** ^1^ Faculty of Medicine and Health, Brain and Mind Centre, The University of Sydney, Sydney, NSW, Australia; ^2^ Faculty of Medicine and Health, Specialty of Child and Adolescent Health, The University of Sydney Children’s Hospital Westmead Clinical School, Sydney, NSW, Australia; ^3^ Department of Psychology, University of Virginia, Charlottesville, VA, United States; ^4^ Kinsey Institute, Indiana University, Bloomington, IN, United States; ^5^ Department of Psychiatry and Behavioural Sciences and Paediatrics, Johns Hopkins University, Baltimore, MD, United States

**Keywords:** Prader-Willi syndrome, oxytocin, vasopressin, behaviour, plasma, saliva

## Abstract

**Background:**

Oxytocin and vasopressin systems are altered in Prader Willi syndrome (PWS). However, investigations into endogenous oxytocin and vasopressin levels as well as clinical trials evaluating the effect of exogenous oxytocin on PWS symptoms have had mixed results. It is also unknown whether endogenous oxytocin and vasopressin levels are associated with certain PWS behaviours.

**Method:**

We compared plasma oxytocin and vasopressin and saliva oxytocin levels in 30 adolescents and adults with PWS to 30 typically developing age-matched controls. We also compared neuropeptide levels between gender and genetic subtypes within the PWS cohort and examined the relationship between neuropeptide levels and PWS behaviours.

**Results:**

While we did not measure a group difference in plasma or saliva oxytocin levels, plasma vasopressin was significantly lower in individuals with PWS compared to controls. Within the PWS cohort, saliva oxytocin levels were higher in females compared to males and individuals with the mUPD compared to the deletion genetic subtype. We also found the neuropeptides correlated with different PWS behaviours for males and females and for genetic subtypes. For the deletion group, higher plasma and saliva oxytocin levels were related to fewer behaviour problems. For the mUPD group, higher plasma vasopressin levels were related to more behaviour problems.

**Conclusion:**

These findings support existing evidence of a vasopressin system defect in PWS and for the first time identify potential differences in the oxytocin and vasopressin systems across PWS genetic subtypes.

## 1 Introduction

Prader Willi syndrome (PWS) is a neurodevelopmental disorder that arises from the absence of expression of paternally inherited imprinted genes in the chromosome 15q11-q13 region (1). In 60-70% of cases, this loss of expression is due to paternal deletion of whole or part of the region; in 20–35% of cases, it is due to maternal uniparental disomy (mUPD) of chromosome 15; and in fewer than 5% of cases, it is due to gene translocation or mutation of the imprinting centre (2, 3). Physical characteristics of PWS include hypotonia, hypogonadism, obesity, dysmorphic facial features, short stature, hypopigmentation, and thick saliva (4). Individuals with PWS can exhibit a range of behaviours, known as the PWS behaviour phenotype, that often begins in childhood and can persist into adulthood (5–8). These behaviours include hyperphagia, temper outbursts, repetitive and ritualistic behaviours, skin-picking, rigidity, and social skill difficulties (5, 9, 10). People with PWS also have an increased risk of developing psychosis (more commonly in the mUPD subtype) and/or depression (more commonly in the deletion subtype), which usually present in adolescence or early adulthood (10, 11). PWS behaviour problems, like hyperphagia, temper outbursts, skin-picking, and psychosis, are the primary cause of morbidity for individuals with PWS and their families (12–14). Unfortunately, there are few if any effective treatments for most PWS behaviour problems. So, there is an urgent need to better understand the nature of and mechanisms underlying these behaviours to inform the development of targeted interventions (10, 15).

Oxytocin (OT) and Arginine Vasopressin (AVP) are two neuropeptides thought to be involved in the PWS phenotype (16, 17). Considered sister-neuropeptides as they share evolutionary origins and differ by only two of the nine amino acids, OT and AVP can act as neurotransmitters, neuromodulators, and hormones (18–20). OT and AVP are primarily produced in the paraventricular nucleus and supraoptic nuclei of the hypothalamus and secreted from the posterior pituitary gland. However, AVP-producing neurons are also found in other limbic regions (21, 22) and a smaller amount of OT and AVP are released peripherally in various tissue (23). The OT receptor and AVP receptors (AVPR1A and AVPR1B) are 85% homologous allowing OT and AVP actions to partly overlap (24, 25).

At the genetic level, people with PWS have reduced expression of the OT receptor gene on chromosome 3p25 in RNA (26); hypomethylation of the OT gene and hypermethylation of 12 of 32 genes in the OT pathway (27). A recent study reported lower methylation in the intron 1 region of the OT receptor gene in DNA blood samples of individuals with PWS compared to age-, sex- and body mass index (BMI) matched controls. The authors further found males with PWS and psychosis showed significantly lower methylation of the OXTR exon region 1 than those without psychosis, suggesting that an OT deficiency in PWS might be associated with the higher rate of psychosis found in PWS (28). Regarding brain function, PWS has long been considered a disorder of the hypothalamus. The hypothalamus plays an integral role in controlling body temperature, hunger, thirst, fatigue, sleep, sexual development, and circadian cycles. All of these are disrupted in PWS (29, 30). Post-mortem studies suggest that compared to typically developing controls, people with PWS have smaller than average hypothalamic periventricular nuclei as well as reduced OT-producing neurons (17); OT mRNA and cells immunoreactive for OT in the hypothalamic paraventricular nuclei (31). These findings suggest a potential OT deficiency in PWS, which is supported by preclinical studies; for a recent summary see (32).

Höybye et al. (33, 34) compared plasma OT levels in people with PWS to a ‘normal range’ level (15 **±** 5 pmol/L) that was established in a previous study. Plasma samples from a previous cohort of typically developing people with obesity were also used in this study therefore, the controls were not age- or sex-matched to the PWS participants (33, 34). Höybye et al. (33, 34) found that plasma OT levels in PWS adults were no different from the ‘normal range’ levels but were significantly lower than plasma OT levels of typically developing people with obesity. The authors suggested that reduced plasma OT levels might be associated with hyperphagia in PWS. In contrast, other studies suggest plasma OT levels (35); and cerebrospinal fluid OT levels (36) are higher in people with PWS compared to typically developing controls. The inconsistency in these findings highlights the need for more research to better understand the nature and potential role of abnormal endogenous OT levels in PWS.

Clinical trials examining the efficacy of exogenous OT on PWS symptomology have also produced mixed findings. Trials that reported positive results suggesting intranasal OT may reduce food-related problematic behaviours (37–39) and improve emotion and behaviour problems and social functioning (37–40) and infant sucking (41). Some trials found that the positive effects of intranasal OT were limited to younger children (38), boys and individuals with the genetic deletion subtype (37). In contrast, two trials found that OT does not produce positive effects on any PWS symptoms (42, 43). One trial found children 12 years of age and older reported significantly more sadness and anger, and less happiness (38) while another found higher doses of OT increased temper tantrums (42), one of the most debilitating behavioural characteristics of PWS (8). Together, these findings suggest that OT may be involved in the PWS behaviour phenotype. However, the inconsistent findings mean the synthetic exogenous OT that has been trialled might not be the most effective treatment and more research is needed to understand the nature of the altered OT system in PWS.

We hypothesised that the increase in temper outbursts observed after the administration of exogenous OT might be explained by the binding of OT to AVP receptors (42). AVP has been shown to play a role in aggressive behaviours (44) and the regulation of emotion and autonomic systems (45). Since people with PWS have reduced OT-producing neurons (17) and their OT receptor gene may be methylated (28) it is possible that they also have a deficit in OT receptors (42). However, even if the OT receptor is not available, exogenous OT might potentially stimulate the AVP receptors, assuming these are still functional in PWS.

Five studies have examined the AVP system in individuals with PWS. The first reported no difference in the number of hypothalamic AVP-producing neurons in people with PWS compared to controls (17). The second found lower CSF AVP levels (36) in females with PWS compared to female controls. However, this difference did not persist when the two males with PWS were removed; that study did not include male controls (36). The third study found the AVP precursor 7B2 was present in two out of five PWS patients. However, the processed AVP was absent in the supraoptic- and paraventricular- nucleus, which the authors suggested might mean that some people with PWS may have a AVP processing deficit (46). Finally, a recent magnetic resonance imaging study found the signal intensity of the ‘bright spot’ in the posterior pituitary gland was negatively correlated with hyperphagia and ASD-like behaviours in adolescents and adults with PWS. The signal intensity is thought to reflect the secretion of the AVP precursor (47). Together, these findings support an AVP system defect in PWS.

We are not aware of a published study that has examined the relationships between endogenous OT and AVP levels and PWS behaviours. This information would help determine what behaviours might be related to OT-AVP system defects and could be used as primary outcome variables in future clinical trials.

The specific aims of the present study are to:

1. Compare plasma OT and AVP and saliva OT levels in individuals with PWS to typically developing age-matched controls.
2. Evaluate whether plasma OT and AVP and saliva OT levels differ across sex, genetic subtype, or the presence of psychosis in individuals with PWS.
3. Examine whether plasma OT levels correlate with saliva OT levels and plasma AVP levels in PWS and controls.
4. Examine the relationship between plasma OT and AVP and saliva OT levels and PWS symptoms.

## 2 Methods

### 2.1 Participants

The study was reviewed and approved by The University of Sydney and the Royal Children’s Hospital Human Research Ethics Committees. Participants were invited into the study *via* a flyer circulated by Australian and international PWS associations and on The University of Sydney website. Participants with PWS were accompanied by a parent or primary caregiver who had known them for at least 12 months. Informed consent was obtained from all participants and the primary caregiver of the person with PWS.

Participants included 30 people with PWS (11 female/19 male) and 30 typically developing age-matched controls (19 female/11 male). Genetic subtype was known for 27 of the 30 participants with PWS: 15 had PWS due to deletion, 11 had PWS due to mUPD, and one had an imprinting centre defect. Six participants with PWS had a history of psychosis, three with mUPD, two with deletion and one with an imprinting centre defect. See **Table 1** for a summary of participant characteristics. Of the 30 participants with PWS, 29 were taking at least one medication. The number of medications ranged from 0-10 with an average of two. The most common medications were antipsychotics (n=11), antidepressants (n=10), and growth hormone (n=8). See **Table 2** for a summary. None of the control participants had a health condition, but one female control was using contraception.

**Table 1:** Participant characteristics, time of blood draw, and neuropeptides measured by group presented as frequency, or mean (SD).

Variables	PWS (n=30)		Controls (n=30)
	Frequency N (%)		
Sex	Male	19 (31.7%)	11 (18.3%)
	Female	11 (18.3%)	19 (31.7%)
Genetic subtype	mUPD	15 (25%)	0
	Deletion	11 (18.3%)	0
	**Mean (SD)**		**Mean (SD)**
Age	22.57_a_ (6.17)		22.43_a_ (3.43)
Height	160.07_a_ (12.06)		170.13_b_ (9.10)
Weight	84.66_a_ (35.18)		64.46_b_ (12.68)
BMI	32.58_a_ (12.73)		22.47_b_ (3.09)
Full IQ	64.14_a_ (15.50)		116.70_b_ (12.19)
Time of blood draw	13.26 (1.85)		13.23 (2.16)
Ln plasma OT	5.14 (0.47)		5.20 (0.51)
Ln plasma AVP	3.80 (0.64)		4.26 (0.58)
Ln saliva OT	3.12 (0.26)		3.12 (0.37)

Values highlighted in blue on the same row not sharing the same subscript are significantly different at p< 0.05.

Cells with no subscript are not included in the test. Tests assume equal variances.

**Table 2:** Medications for participants with PWS, frequency (%).

Medication	Total (n=30)	Male (n=19)	Female (n=11)
Antipsychotic	11 (37%)	8 (42%)	3 (27%)
SSRI	10 (33%)	6 (32%)	4 (36%)
Levothyroxine	7 (23%)	4 (21%)	3 (27%)
Testosterone	7 (23%)	7 (37%)	–
Growth Hormone	8 (22%)	6 (32%)	2 (18%)
Oestrogen	5 (17%)	–	5 (46%)
Metformin	5 (17%)	4 (21%)	1 (9%)
Gliclazide	4 (13%)	2 (11%)	2 (18%)
Progesterone	2 (7%)	–	2 (19%)

### 2.2 Measures

The intelligence test was completed by people with PWS and typically developing controls. All other measures focused on PWS behaviours and thus were only completed for the PWS cohort by a parent or primary caregiver.

#### 2.2.1 Intelligence

The Wechsler Abbreviated Scale of Intelligence Second Edition (WASI-II) (48) was used to assess intelligence quotient (IQ). People who had a Wechsler IQ test administered within the last two years did not have to do this test if data from the previous test were available.

#### 2.2.2 Emotion and behaviour problems

The Developmental Behaviour Checklist (DBC) Primary Carer (49) and Adult Versions (49) were used to assess emotion and behaviour problems as reported by parents of people with PWS. The DBC is an informant measure that has been successfully used in a number of PWS studies (6, 7, 9, 42, 50). The Total Behaviour Problem Score (TBPS) gives an overall measure of emotion and behaviour disturbance, the subscale scores describe domains of disturbance, and the individual items can give a fine-grained indication of specific problems. The DBC has high inter-rater reliability (ICC =0.80), high internal consistency (0.941) and high concurrent validity and has been tested with other measures of behaviour disturbance. The sensitivity to change of these measures has been documented (51). The DBC-A subscales were used in the present study, subscale item examples are provided in the **Supplementary Material** to provide an indication of the constructs they measure.

#### 2.2.3 Hyperphagia

The Hyperphagia Food Questionnaire for Clinical Trials (HQ-CT) was used to measure eating behaviours (52). Based on the Dykens Hyperphagia (53); the HQ-CT includes nine items that assess the severity of hyperphagic behaviours specific to PWS on a 5-point Likert scale and are summed for a total score, with higher scores indexing more hyperphagic symptoms.

#### 2.2.4 Emotion recognition

The Ekman Emotion Recognition Task is the most widely used and validated series of photographs in facial expression research (54). This test was used to assess participants’ abilities to recognise emotions from faces. This test comprises 60 photographs of faces posed by actors, each photograph depicting one of the basic emotions: happiness, sadness, anger, fear, surprise, and disgust. This measure has previously been used with individuals with PWS (55).

#### 2.2.5 Sleep

The Epworth sleepiness scale (ESS) is a simple 8-item measure widely used in sleep research including in people with intellectual disabilities and PWS (42, 56, 57). It has been shown to have good content validity when used with people with PWS (58). The primary caregiver of the person with PWS completed the ESS.

### 2.3 Procedure

The study was conducted at The University of Sydney’s Brain and Mind Centre and the Royal Children’s Hospital in Melbourne.

#### 2.3.1 Plasma collection

Participants underwent a blood draw. Approximately 2000 µl of blood was collected in chilled glass tubes containing disodium EDTA and kept in ice. After collection, the blood sample was centrifuged at 3000-3500 rpm for ten min at 4°C. The plasma (supernatant) was collected (400 µl per aliquot), and aliquoted into two microcentrifuge tubes (1.5 ml Eppendorf tubes) immediately and stored at -80°C (for long term) until assay.

#### 2.3.2 Saliva collection

Participants’ saliva samples were collected using a Salivette. Participants were asked to leave the Salivette swab in their mouth for two minutes without chewing. The swab was then sealed, labelled, and stored for analysis. Salivettes were ice-chilled for up to 1 hour before being centrifuged at 4°C at 1500 × g for 15 minutes. The liquid samples were stored at −80°C.

#### 2.3.3 Plasma and saliva processing

To measure the concentrations of OT and AVP, highly sensitive Enzyme Immunoassay kits (EIA; Arbor Assays LLC., Ann Arbor, Michigan, USA) were used. The EIA has a minimal detection rate of 16.38 pg/mL for OT and 4.096 pg/mL for AVP. The EIA has minimal cross-reactivity for other neuropeptides. To ensure the reliability of the assays, all samples were run at the same time by an investigator blind to the origins of the samples. All but one of each of the plasma coefficients of variance were less than 14.4 (m=6.03) for OT and 18.4 (m=3.5) for AVP in intra-assays, and less than 4.97 for OT and 1.42 for AVP in inter-assays. Removing the two samples that had higher coefficients made no difference to the findings, so they were retained. Similarly, the saliva CV were less than 8.88 for OT in intra-assays and less than 3.88 for OT in inter-assays. The samples were not extracted as previous research has shown that accurate measurement of OT in human blood plasma can be obtained without extraction (59, 60). Additional information on extracted vs. non-extracted plasma OT measurement can be found in the study by Plasencia et al. (61).

### 2.4 Statistical analyses

Analyses were conducted using IBM SPSS Statistics 28 for Windows. Two-tailed unpaired independent t-tests were conducted to determine the between-group differences (PWS vs. typically developing controls) for participant demographic characteristics. Categorical variables such as sex and genetic subtype are presented as frequencies while continuous variables are presented as mean (SD). Known confounders of endogenous OT levels include sex, time of day, age and menstrual cycle variation (62, 63). We controlled for all but menstruation as only 3/11 females with PWS had a menstrual cycle, which is not uncommon in females with PWS (64).

Comparisons of plasma and saliva neuropeptide levels between categorical variables like group allocations (PWS vs. control), sex (male vs. female) genetic subtype (mUPD vs. deletion), and psychosis (Yes vs. No) were analysed by building multifactorial one-way analysis of covariance (ANCOVA) models using the general linear model (GLM) function in SPSS. The GLM was chosen to ascertain if a combination of the categorical predictor variables explains the variability in neuropeptide levels. Age and time of blood draw were included as covariates to account for possible confounding effects. For significant results from the GLM, pairwise comparisons were made using Bonferroni as a *post hoc* test to determine if a significant difference was present in each group. ANCOVA adjusts for continuous covariates so there is a distinctive assessment of the impacts of discrete predictors. The significance level was set at p<0.05 but given the exploratory nature of the study, near-significant trends at p<0.1 were highlighted in the results.

Pearson’s correlation coefficients were used to correlate neuropeptides against each other in the entire participant cohort followed by correlation by subgroups including PWS only, typically developing control group only, the male and female cohort respectively as seen in **Table 3**.

**Table 3:** Independent samples t-test results of neuropeptides and time of blood draw across sex, genetic subtype, and psychosis in PWS group only.

Variables	PWS ONLY					
	Sex		Genetic Subtype		Psychosis	
	Male	Female	Deletion	mUPD	Yes	No
	Mean (SD)					
Ln Plasma OT	5.09 (0.55)	5.22 (0.29)	5.19 (0.39)	5.23 (0.39)	5.10 (0.45)	5.16 (0.49)
Ln Plasma AVP	3.77 (0.66)	3.84 (0.62)	3.89 (0.72)	3.81 (0.58)	4.02 (0.52)	3.75 (0.68)
Ln Saliva OT	3.06 (0.24)	3.21 (0.28)	3.11 (0.25)	3.22 (0.32)	3.33 (0.38)	3.10 (0.24)
Time of Blood Draw	13.10 (1.88)	13.54 (1.86)	13.47 (1.35)	12.82 (1.99)	14.40 (2.41)	12.90 (1.61)

There was no significant difference.

Correlations were also conducted for neuropeptides against variables representing emotion and behaviour problems including, TBPS from the DBC, single subscale items from the DBC, Ekman Emotion Recognition Task, ESS, and HQ-CT. Mean item scores were used to combine the TBPS and shared DBC-P and DBC-A subscales. However, some DBC-P and A subscales differ slightly between the two measures. We used the DBC-A subscale, so the subscales that are unique to the DBC-A only included data from adults with PWS and have a slightly smaller sample size.

## 3 Results

The natural log transformations (Ln) of neuropeptides were used for statistical analyses as tests of normality resulted in non-normally distributed (skewed) outputs. All other respective data assumptions were met for the parametric test conducted, including normality, constant variance, and parallel lines.

### 3.1 Participant characteristics

Independent samples t-test revealed that age was similar across those with PWS and controls. Weight and BMI were significantly higher in the PWS group while height and IQ were higher in the typically developing group (**Table 1**). AVP was significantly different (t= -2.93, p= 0.005) between groups, i.e., lower in PWS compared to typically developing controls (**Table 1**). The t-test results for neuropeptides and time of blood draw across sexes, genetic subtypes and presence of psychosis showed no significant differences (**Table 3**). There were 14 participants with PWS taking sex (testosterone, oestrogen and/or progesterone) and/or thyroid medication, which holds the potential to influence neuropeptide levels. Differences in AVP levels between people with PWS and controls remained when these participants were removed. The sample size was too small to conduct comparisons within the PWS participants (sex, genetic subtype, and presence of psychosis) if levels from these 14 were removed.

### 3.2 Comparing neuropeptides in the full cohort across group allocations and sex with covariates

**Figure 1:**
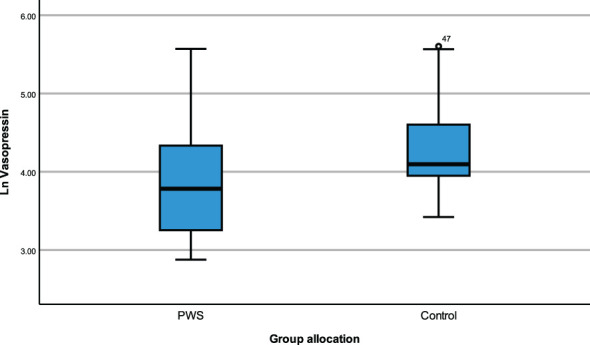
Simple boxplot of vasopressin by group of allocation (PWS and control).

ANCOVA models were developed to compare the impact of group allocation (PWS vs. control) and sex (male vs female) categories on the neuropeptides i.e., plasma OT, plasma AVP and saliva OT levels while controlling for age and the time of blood draw as covariates. There were no significant differences in plasma OT levels between PWS and controls [F (1,55) = 0.184, p= 0.669] or between males and females [F (1,55) = 0.037, p = 0.848]. Non-significant results were also found for saliva OT levels between sex and group allocation at [F (1,51) = 3.729, p = 0.059] and [F (1,51) = 0.266, p = 0.608], respectively. However, for the plasma AVP neuropeptide, levels were significantly lower [F (1,55) = 6.564, p = 0.013)] in PWS (M = 3.142, SE= 0.064) compared to controls (M = 3.096, SE = 0.060) as depicted in the boxplot (**Figure 1**). There were no significant differences in AVP between males and females [F (1,55) = 1.058, p = 0.308]. See **Table 4**.

**Table 4:** General Linear model/Univariate analyses of covariance of neuropeptides across fixed factor variables controlling for age and time of blood draw as covariates.

Cases	Neuropeptides	Categorical Variables		Statistics	
		Subgroup 1	Subgroup 2	ANCOVA adjusted for Age and Time of Blood Draw	
		Mean ± SE	Mean ± SE	F	p-value
**Full Cohort**	Ln Plasma OT				
	Group Allocation	PWS	Control		
		5.143 (0.093)	5.201 (0.093)	0.184	0.669
	Sex	Male	Female		
		5.159 (0.094)	5.185 (0.094)	0.037	0.848
	Ln Plasma AVP				
	Group Allocation	PWS	Control		
		3.142 (0.064)	3.096 (0.060)	6.564	0.013
	Sex	Male	Female		
		3.032 (0.063)	3.206 (0.062)	1.058	0.308
	Ln Saliva OT				
	Group Allocation	PWS	Control		
		3.142 (0.064)	3.096 (0.060)	0.266	0.608
	Sex	Male	Female		
		3.032 (0.063)	3.206 (0.062)	3.729	0.059
**PWS only**	Ln Plasma				
	Genetic Subtype	mUPD	Deletion		
		5.259 (0.133)	5.190 (0.109)	0.163	0.691
	Sex	Male	Female		
		5.182 (0.100)	5.267 (0.140)	0.243	0.627
	Ln Plasma AVP				
	Genetic Subtype	mUPD	Deletion		
		3.880 (0.221)	3.872 (0.182)	0.001	0.977
	Sex	Male	Female		
		3.826 (0.167)	3.926 (0.234)	0.121	0.732
	Ln Saliva OT				
	Genetic Subtype	mUPD	Deletion		
		3.319 (0.083)	3.107 (0.059)	4.398	0.050
	Sex	Male	Female		
		3.087 (0.059)	3.340 (0.081)	6.543	0.020
**PWS only**	Ln Plasma OT				
	Psychosis	Yes	No		
		4.972 (0.219)	5.218 (0.100)	1.036	0.319
	Sex	Male	Female		
		5.016 (0.134)	5.175 (0.165)	0.765	0.390
	Ln Plasma AVP				
	Psychosis	Yes	No		
		3.912 (0.312)	3.788 (0.143)	0.129	0.722
	Sex	Male	Female		
		3.816 (0.192)	3.884 (0.235)	0.070	0.794
	Ln Saliva OT				
	Psychosis	Yes	No		
		3.319 (0.083)	3.107 (0.059)	2.642	0.120
	Sex	Male	Female		
		3.087 (0.059)	3.340 (0.081)	5.492	0.030

Values highlighted in blue, and green are significant or near significant at p < 0.05 and p < 0.1, respectively.

### 3.3 Comparing neuropeptides in the PWS group across genetic subtypes and sex with covariates

**Figure 2:**
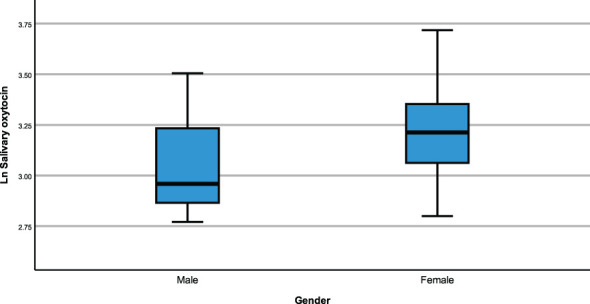
Simple boxplot of salivary oxytocin by gender (male and female) in the PWS group.

**Figure 3:**
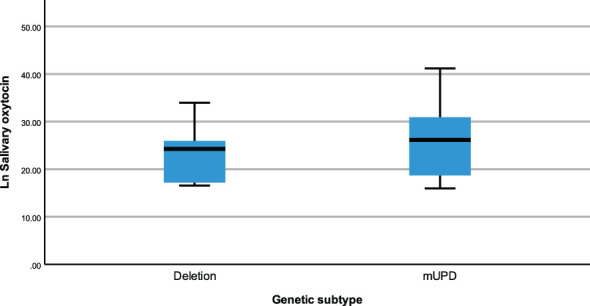
Simple boxplot of salivary oxytocin by genetic subtype (deletion and mUPD) in the PWS group.

The same modelling process was carried out to examine whether neuropeptides differed across genetic subtypes and sex within the PWS cohort. There were no differences recorded between plasma OT and AVP levels across both groups (genetic subtype and sex). However, saliva OT was significantly higher [F (1,17) = 6.543, p= 0.020] in females with PWS (M = 3.340, SE = 0.081) compared to males with PWS (M = 3.087, SE = 0.059) (**Figure 2**). Similarly, significant saliva OT levels F (1,17) = 4.398, p = 0.050] were found in the genetic subtype with higher levels in the mUPD group (M = 3.319, SE = 0.083) compared to deletion (M = 3.107, SE = 0.059) also shown in the boxplot, **Figure 3**. The effect size was evaluated from the partial ETA squared result of 0.278 indicating that a 27.8% change/variance in saliva OT can be accounted for by sex. Similarly, the effect size from the partial ETA squared result for the genetic subtype was 0.206, meaning a 20.6% change in saliva OT was attributed to the genetic subtype. See **Table 4**.

### 3.4 Comparing neuropeptides in the PWS group across psychosis and sex with covariates

An ANCOVA model was generated to examine whether neuropeptides differed between those with and without psychosis and sex categories focusing on the PWS group only and controlling for age and time of blood draw as covariates. Results show no significant differences in plasma OT and plasma AVP levels across both psychosis and sex groups. In saliva OT, there were significant results for sex [F (1,20) = 5.492, p = 0.030] with females (M = 3.340, SE = 0.081) having higher level of saliva OT than males (M = 3.087, SE = 0.059). The effect size was evaluated from the partial ETA squared result of 0.215 indicating that a 21.5% change/variance in saliva OT can be attributed to sex.

### 3.5 Relationship between OT and AVP levels

**Figure 4:**
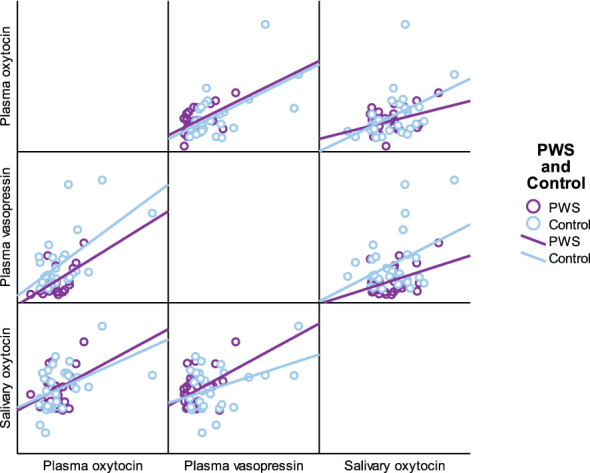
Scatterplot matrix of neuropeptides by group allocation (PWS and control).

**Figure 5:**
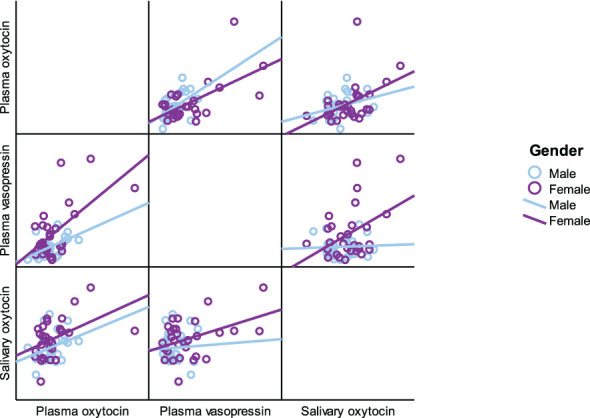
Scatterplot matrix of neuropeptides by gender (male and female).

As presented in **Table 5**, plasma OT had a significant positive correlation with plasma AVP and saliva OT levels in the full cohort (r^2^ = 0.550, p < 0.001, r^2^ = 0.411, p = 0.002), typically developing control group (r^2^ = 0.561, p = 0.001, r^2^ = 0.518, p = 0.003) and female group (r^2^ = 0.485, p = 0.007, r^2^ 0.483 = 0.008), respectively. Scatterplots of neuropeptides by group allocation and gender are shown in **Figures 4**, **5**. However, there were only significant correlations between plasma OT and plasma AVP for PWS (r^2^ = 0.574, p < 0.001) and male-only groups (r^2^ = 0.636, p < 0.001). All other correlations across neuropeptides were not significant but there was a positive trend between plasma AVP and saliva OT (r^2^ = 0.224, p = 0.097) in the full cohort.

**Table 5:** Neuropeptide correlation for all participants, PWS and control separately and males and females separately.

	All COHORT			PWS			CONTROL			MALES			FEMALES		
	Plasma OT	Plasma AVP	Saliva OT	Plasma OT	Plasma AVP	Saliva OT	Plasma OT	Plasma AVP	Saliva OT	Plasma OT	Plasma AVP	Saliva OT	Plasma OT	Plasma AVP	Saliva OT
Plasma OT	1			1			1			1			1		
	60			30			30			30			30		
	Plasma AVP	.550^**^	1		.574^**^	1		.561^**^	1		.636^**^	1		.485^**^	1
		<.001			<.001			.001			<.001			.007	
		60	60		30	30		30	30		30	30		30	30
Saliva OT	.411^**^	.224	1	.235	.290	1	.518^**^	.231	1	.307	-.030	1	.483^**^	.273	1
	.002	.097		.247	.150		.003	.219		.119	.882		.008	.152	
	56	56	56	26	26	26	30	30	30	27	27	27	29	29	29

LN OT, natural log-transformed plasma OT; LN AVP, natural log-transformed plasma vasopressin; LN OTS, natural log-transformed saliva OT. Each cell provides Pearson r^2^, p-value, and the number of participants (n), respectively. Highlighted in blue is a statistically significant trend at <0.05. Cells highlighted in blue with ** are statistically significant at <0.01.

### 3.6 Relationship between OT and AVP levels and behaviour

Given that differences were found in neuropeptide levels across sex and genetic subtype within the PWS cohort, we examined the association between neuropeptide levels and behaviours separately for males and females and for deletion and mUPD groups, see **Table 6**. Given the number of analyses (9 behaviours for each sex and genetic subtype) and the small sample size (n=30), these findings must be interpreted with caution.

**Table 6:** Relationships between plasma OT, AVP and saliva OT levels and symptoms in the PWS groups.

	ALL PWS			PWS MALES			PWS FEMALES			PWS Deletion			PWS UPD		
	OT	AVP	OTS	OT	AVP	OTS	OT	AVP	OTS	OT	AVP	OTS	OT	AVP	OTS
DBC MIS TBPS	-0.34	0.05	0.00	-0.58**	-0.10	-0.21	0.41	0.34	0.31	-0.47	-0.30	-0.15	0.19	0.71^*^	0.15
	0.07	0.79	0.99	0.009	0.68	0.43	0.21	0.31	0.38	0.080	0.285	0.601	0.585	0.015	0.717
	30	30	26	19	19	16	11	11	10	15	15	14	11	11	8
DBC MIS Disruptive	-0.38^*^	-0.03	-0.08	-0.46*	-0.22	-0.03	-0.19	0.32	-0.12	-0.54^*^	-0.38	0.01	-0.07	0.41	-0.03
	0.04	0.90	0.69	0.05	0.36	0.93	0.58	0.33	0.75	0.040	0.167	0.986	0.847	0.209	0.941
	30	30	26	19	19	16	11	11	10	15	15	14	11	11	8
DBC MIS Communication	-0.17	0.18	-0.01	-0.34	0.13	-0.25	0.49	0.31	0.42	-0.26	0.00	-0.03	0.08	0.59	-0.04
	0.37	0.34	0.98	0.16	0.61	0.34	0.12	0.35	0.22	0.349	0.992	0.925	0.810	0.056	0.922
	30	30	26	19	19	16	11	11	10	15	15	14	11	11	8
DBC MIS Self-absorbed	-0.08	0.20	0.05	-0.27	0.18	-0.21	0.76^**^	0.30	0.48	-0.07	0.03	-0.47	0.29	0.65^*^	0.38
	0.67	0.28	0.80	0.27	0.45	0.44	0.007	0.38	0.16	0.800	0.923	0.088	0.389	0.029	0.358
	30	30	26	19	19	16	11	11	10	15	15	14	11	11	8
DBC MIS Depressive	-0.13	0.18	0.47^*^	-0.38	-0.04	0.12	0.43	0.46	0.78	-0.39	-0.10	0.56	0.33	0.71^*^	0.37
	0.58	0.44	0.05	0.16	0.89	0.72	0.34	0.30	0.07	0.206	0.749	0.072	0.418	0.047	0.539
	22	22	18	15	15	12	7	7	6	12	12	11	8	8	5
DBC MIS Social relating	-0.21	0.13	0.33	-0.57*	-0.08	-0.34	0.45	0.39	0.82^**^	-0.38	-0.14	0.31	0.29	0.72^*^	0.35
	0.26	0.51	0.11	0.01	0.73	0.19	0.17	0.24	0.004	0.167	0.609	0.276	0.386	0.013	0.390
	30	30	26	19	19	16	11	11	10	15	15	14	11	11	8
Epworth daytime sleepiness scale	-0.08	0.37^*^	0.09	-0.15	0.24	-0.16	0.13	0.60*	0.42	-0.05	0.39	0.21	0.14	0.53	0.04
	0.68	0.05	0.67	0.55	0.33	0.55	0.70	0.05	0.23	0.849	0.151	0.474	0.688	0.093	0.926
	30	30	26	19	19	16.	11	11	10	15	15	14	11	11	8
Hyperphagia questionnaire total score	-0.23	0.16	0.13	-0.51*	-0.12	-0.06	0.14	0.56	0.06	-0.41	-0.09	0.03	0.49	.790^*^	0.61
	0.24	0.41	0.54	0.03	0.63	0.81	0.71	0.09	0.88	0.124	0.763	0.907	0.184	0.011	0.146
	28	28	25	18	18	16.	10	10	9	15	15	14	9	9	7
Ekman emotion recognition	-0.02	0.01	-0.18	0.18	0.03	-0.14	-.61^*^	-0.05	-0.37	0.10	-0.04	0.12	-0.38	-0.17	-0.68
	0.92	0.95	0.39	0.49	0.89	0.62	0.02	0.89	0.29	0.715	0.887	0.677	0.314	0.662	0.062
	28	28	26	17	17	16.	11	11	10	15	15	14	9	9	8

Highlighted in green is a nearly statistically significant trend at <0.1; highlighted in blue is statistically significant at <0.05. Each cell provides Pearson r^2^, p-value, and the number of participants (n), respectively. Correlation is significant at the 0.01 level (2-tailed)^**^. Correlation is significant at the 0.05 level (2-tailed)^*^. Full table is available in the **Supplementary Material** (**Table 3**). DBC MIS TBPS, Developmental Behaviour Checklist Mean Item Score Total Behaviour Problem Score.

#### 3.6.1 PWS cohort

The relationship between OT and AVP levels and behaviour was examined within the PWS group only. For the entire PWS cohort, we found higher levels of plasma OT level were related to lower disruptive behaviours (r^2^ = -0.38, p = 0.04) as well as DBC TBPS, although the latter was not statistically significant. Plasma AVP levels positively correlated with daytime sleepiness (r^2^ = 0.37, p = 0.05). Saliva OT also positively correlated with depressive symptoms (r^2^ = 0.47, p = 0.05).

#### 3.6.2 PWS males

For PWS males, significant correlations were recorded between plasma OT with the strongest negative correlation in the DBC TBPS overall (r^2^ = -0.58, p= 0.009) followed by social relating difficulties (r^2^ = -0.57, p= 0.01), symptoms of hyperphagia (r^2^ = -0.51, p = 0.03) and disruptive behaviour scales (r^2^ = -0.46, p= 0.05). This means that increased plasma OT in PWS males was related to decreased behaviour problems, disruptive behaviour, social relating difficulties and hyperphagia.

#### 3.6.3 PWS females

For PWS females, significant correlations among specific PWS symptoms were observed across all three neuropeptides (plasma OT and AVP and saliva OT). Plasma OT was positively correlated with being self-absorbed (r^2^ = 0.76, p= 0.007) and negatively related to emotion recognition abilities (r^2^ = -0.61, p= 0.02). Plasma AVP positively correlated with daytime sleepiness (r^2^ = 0.60, p = 0.05). Finally, saliva OT was positively correlated with social relating difficulties (r^2^ = 0.82, p = 0.004).

#### 3.6.4 Deletion subtype

**Figure 6:**
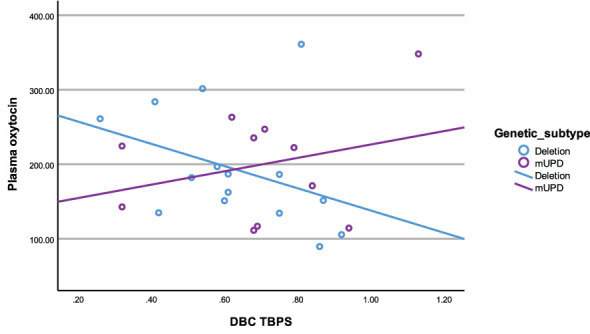
Scatterplot of plasma oxytocin and DBC TBPS by genetic subtype (deletion and mUPD).

In the deletion group, there was a negative correlation between plasma OT and disruptive behaviour (r^2^ = -0.54, p= 0.04). All other correlations in the deletion subtype were negative and trending towards significance between plasma OT and TBPS (**Figure 6**) as well as saliva OT and self-absorbed and depressive behaviour but did not reach statistical significance.

#### 3.6.5 mUPD subtype

**Figure 7:**
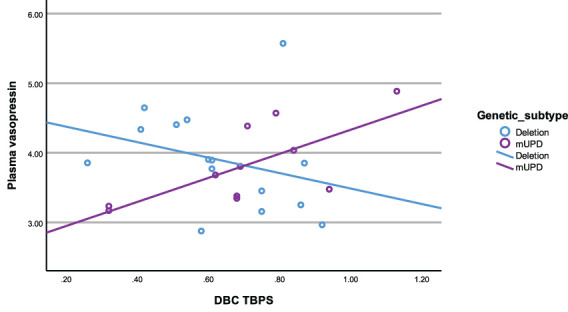
Scatterplot of plasma vasopressin and DBC TBPS by genetic subtype (deletion and mUPD).

In the mUPD genetic subtype, although there were no significant correlations recorded against plasma OT and any PWS symptom, plasma AVP was significantly and positively correlated with five PWS symptoms including TBPS (**Figure 7**), hyperphagia, social relating difficulties and self-absorbed and depressive behaviours (r^2^ = 0.71, p= 0.015; r^2^ = 0.79, p= 0.011; r^2^ = 0.72, p= 0.013; r^2^ = 0.65, p= 0.029; r^2^ = 0.71, p= 0.047, respectively). There was also a trend towards significance for a negative correlation between saliva OT and emotion recognition.

## 4 Discussion

In the present study, we examined differences in plasma OT and plasma AVP and saliva OT levels in people with PWS and age-matched typically developing controls and the relationship between these neuropeptides and PWS behaviours. People with PWS had lower plasma AVP levels than controls. No difference was found in plasma or saliva OT levels between the cohorts. For people with PWS, there was no difference in neuropeptide levels between those with and without psychosis. However, saliva OT levels were lower in people with the deletion genetic subtype compared to the mUPD genetic subtype and in males compared to females.

We found plasma AVP levels were lower in people with PWS compared to age-matched controls. Our findings support the existing literature, which suggests a potential AVP system defect in PWS (36, 46, 47). Studies that have compared AVP levels in individuals with autism spectrum disorder (ASD) to typically developing controls have reported mixed results (65–69). The sparsity of studies and variability in methodology and findings make it difficult to draw conclusions about the role of AVP in ASD (70) However, a randomised-controlled trial (RCT) of intranasal AVP reported improvements in social abilities, anxiety, and some repetitive behaviours in those taking AVP compared to placebo (71). The only other trial of an AVP product in ASD was an RCT of Balovaptan, a selective AVP 1_a_ receptor antagonist. This large multi-centre study found no improvements (72). AVP has not been trialled in individuals with PWS. However, centrally administered AVP has been shown to “rescue” social deficits in *Magel2-deficient* mice (73). *Magel*2 is one of the genes that is reduced in expression in the PWS critical region of chromosome 15.

The findings from two meta-analyses suggest endogenous OT (plasma, urine, or saliva) levels are lower in children with ASD compared to typically developing controls but that this difference is not present in adolescents and adults with ASD (74, 75). Age differences might explain why our findings differed from a study that reported plasma OT levels to be higher in children with PWS (mean age 8.2 years; SD 2) compared to controls (35) but aligned with a study conducted with adults with PWS that reported similar rates of plasma OT with a ‘normal’ range (33). Our findings also differed from a study that reported higher CSF OT levels in adolescents and adults with PWS compared to typically developing controls (36), which might be due to differences in plasma and CSF. A meta-analysis of 17 studies found that plasma and CSF OT levels did not correlate at baseline but did after experimentally inducing stress. However, this meta-analysis did not control for covariates like age and sex and the methodologies varied greatly across studies, including both humans and other species (76).

Recent studies have found stronger correlations in OT levels between CSF and saliva than between CSF and plasma (77, 78), suggesting that saliva might be a better biomarker of central OT levels. Our study is the first to examine saliva OT levels in individuals with PWS, we found no difference to typically developing controls. However, saliva OT levels were lower in people with PWS due to deletion compared to the mUPD genetic subtype and in males compared to females with PWS. These differences were not found in our plasma samples or plasma samples collected from children with PWS (35). We also found that for those with deletion, PWS behaviours positively correlated with plasma and saliva OT but not with plasma AVP. Conversely, for those with mUPD behaviours negatively correlated with plasma AVP but not plasma OT. These findings are discussed in more detail below. Interestingly, the longest clinical trial of intranasal OT conducted in PWS reported more significant improvements in eating and socialising behaviours in children with deletion than mUPD subtype and in males compared to females, which aligns with our findings (37). Individuals with mUPD tend to have more social communication difficulties and are more likely to meet the criteria for ASD than people with PWS due to deletion (79). Based on these behavioural differences and the known decrease in OT in children with ASD (74, 75), one might expect the PWS mUPD cohort to have lower OT levels than those with deletion. It might be that the higher rate of ASD-like behaviours in those with mUPD is associated with AVP system defects rather than OT. Future studies comparing saliva OT levels across genetic subtypes are needed to verify our findings.

Research conducted within the typically developing population has also reported lower OT levels in males compared to females (80, 81). A meta-analysis of studies conducted with people with ASD found that lower OT levels in children with ASD compared to typically developing controls were only present in males (75). However, studies conducted with adult psychiatric populations, such as major depressive disorder and obsessive-compulsive disorder reported no difference in OT levels across sexes (81). It might be that reduced OT in males with PWS is not related to the syndrome but rather part of typical development.

A recent study reported lower methylation of the OXTR exon region 1 in males with PWS with psychosis compared to those without psychosis (28). We found no difference in OT or AVP levels between people with PWS with and without psychosis, however, only five individuals with PWS in our cohort had psychosis. So, our sample size might have been too small to detect a difference.

We examined the relationship between neuropeptides and PWS symptoms. Given differences were found in saliva OT levels across sex and genetic subtype, we examined the relationship between neuropeptides in males and females and the deletion and mUPD genetic subtype separately. For males, higher plasma OT correlated with fewer behaviour problems, including disruptive behaviour, social relating (ASD-like) difficulties and hyperphagia. For females, higher OT was associated with being self-absorbed and having difficulty with emotion recognition. These findings suggest OT and AVP might be related to different behaviour and social skill difficulties across sexes in PWS and highlight the need to identify sex differences when examining OT-AVP system abnormalities in PWS or trialling related interventions.

In people with the deletion subtype of PWS, higher plasma OT correlated with fewer behaviour problems and higher saliva OT correlated with fewer depressive and self-absorbed difficulties. There was no relationship with AVP. For people with the mUPD subtype the reverse was true, higher AVP correlated with higher behaviour problems, including depressive, communication, social relating, and self-absorbed difficulties as well as with higher rates of hyperphagia. There was no relationship with plasma OT and only a nearly significant negative correlation with saliva OT and emotion recognition. These findings suggest that for PWS, some behaviour problems might relate to OT system abnormalities for those with deletion and AVP system abnormalities for those with mUPD. Future studies are needed to validate our findings.

There are several study limitations worth mentioning. First, peripheral neuropeptide levels can differ from those of central levels (63, 76). While some studies suggest saliva OT might be a better biomarker of central OT than plasma (77, 78), more research is needed to confirm this hypothesis. Second, 14 participants with PWS in the current study were taking sex (testosterone, oestrogen and/or progesterone) and/or thyroid medication that could alter neuropeptide levels. Differences in AVP levels between people with PWS and controls remained significant when these participants were removed. The sample size was too small to conduct comparisons within the PWS participants if levels from these 14 were removed. While children with PWS are given growth hormones from a young age, sex hormones are typically prescribed post-puberty, so studies conducted with children could help control for this confound. Third, some authors have recommended sample extraction prior to assay for measuring OT; as unextracted samples provide higher levels than those that are extracted (82). As explained elsewhere, OT has unique properties that make this molecule both biologically active and difficult to measure (60, 83). However, recent research suggests that this discrepancy in estimates of OT may result from the removal of bound OT during the extraction step (60, 84). Whether the bound or unbound OT is more biologically active is unclear. However, several recent studies comparing functional relationships between extracted versus unextracted samples have suggested that stronger relationships between OT and behaviour are detected in measurements of unextracted plasma samples (85, 86).

Finally, this was an exploratory study with several analyses and relatively small sample size, so our findings, particularly the correlation analyses between the neuropeptides and behaviour, must be interpreted with caution.

## 5 Conclusions

This is the first study to examine saliva OT levels in PWS and to examine the relationship between endogenous OT and AVP and PWS behaviour. We found plasma AVP was significantly lower in individuals with PWS compared to age-matched controls. Within the PWS cohort, saliva OT levels were lower in males compared to females and individuals with the deletion vs mUPD genetic subtype. We also found that the neuropeptides correlated with different PWS behaviours for males and females and for genetic subtypes. For the deletion group, higher plasma and saliva OT levels were related to fewer behaviour problems and for mUPD higher plasma AVP levels were related to more PWS behaviours. Our findings highlight the need to consider sex and genetic subtype differences in future investigations of these neuropeptides.

## Data availability statement

The datasets presented in this article are not readily available because of ethical and privacy restrictions. Requests to access the datasets should be directed to the corresponding author.

## Ethics statement

The studies involving human participants were reviewed and approved by The University of Sydney and the Royal Children’s Hospital Human Research Ethics Committees. Written informed consent to participate in the study was provided by the participants and the legal guardian/next of kin for participants with PWS.

## Author contributions

LR, SE, CC, and JH designed the research and obtained financial support. LR obtained ethics and governance approval, recruited participants, and collected data. HPN analysed the plasma and saliva samples. LR and JA analysed the data. LR, HN, and JA wrote the manuscript. All authors contributed to the article and approved the submitted version.

## References

[1] Smith A. The diagnosis of prader–willi syndrome. J paediatrics Child Health (1999) 35(4):335–7. doi: 10.1046/j.1440-1754.1999.00396.x 10457285

[2] Butler MG Hartin SN Hossain WA Manzardo AM Kimonis V Dykens E. Molecular genetic classification in prader-willi syndrome: a multisite cohort study. J Med Genet (2019) 56(3):149–53. doi: 10.1136/jmedgenet-2018-105301 PMC7387113 29730598

[3] Cassidy SB Schwartz S Miller JL Driscoll DJ. Prader-willi syndrome. Genet Med (2012) 14(1):10–26. doi: 10.1038/gim.0b013e31822bead0 22237428

[4] Holm VA Cassidy SB Butler MG Hanchett JM Greenswag LR Whitman BY. Prader-willi syndrome: consensus diagnostic criteria. Pediatrics (1993) 91(2):398–402. doi: 10.1542/peds.91.2.398 8424017 PMC6714046

[5] Holland A Whittington J Butler J Webb T Boer H Clarke D. Behavioural phenotypes associated with specific genetic disorders: evidence from a population-based study of people with prader-willi syndrome. psychol Med (2003) 33(1):141–53. doi: 10.1017/S0033291702006736 12537045

[6] Rice LJ Gray KM Howlin P Taffe J Tonge BJ Einfeld SL. The developmental trajectory of disruptive behavior in down syndrome, fragile X syndrome, prader–willi syndrome and williams syndrome. Am J Med Genet Part C: Semin Med Genet (2015) 169(2):182–7. doi: 10.1002/ajmg.c.31442 25983069

[7] Rice LJ Gray KM Howlin P Taffe J Tonge BJ Einfeld SL. The developmental trajectory of self-injurious behaviours in individuals with prader willi syndrome, autism spectrum disorder and intellectual disability. Diseases (2016) 4(1):9. doi: 10.3390/diseases4010009 28933389 PMC5456304

[8] Rice LJ Woodcock K Einfeld SL. The characteristics of temper outbursts in prader–willi syndrome. Am J Med Genet Part A (2018) 176(11):2292–300. doi: 10.1002/ajmg.a.40480 30289600

[9] Einfeld SL Smith A Durvasula S Florio T Tonge BJ. Behavior and emotional disturbance in prader-willi syndrome. Am J Med Genet (1999) 82(2):123–7. doi: 10.1002/(SICI)1096-8628(19990115)82:2<123::AID-AJMG4>3.0.CO;2-C 9934974

[10] Schwartz L Caixàs A Dimitropoulos A Dykens E Duis J Einfeld S. Behavioral features in prader-willi syndrome (Pws): consensus paper from the international pws clinical trial consortium. J Neurodev Disord (2021) 13(1):1–13. doi: 10.1186/s11689-021-09373-2 34148559 PMC8215770

[11] Whittington J Holland A. A review of psychiatric conceptions of mental and behavioural disorders in prader-willi syndrome. Neurosci Biobehav Rev (2018) 95:396–405. doi: 10.1016/j.neubiorev.2018.10.006 30392879

[12] Bos-Roubos A Wingbermühle E Biert A de Graaff L Egger J. Family matters: trauma and quality of life in family members of individuals with prader-willi syndrome. Front Psychiatry (2022) 13:897138. doi: 10.3389/fpsyt.2022.897138 35836666 PMC9273751

[13] Dykens EM Roof E Hunt-Hawkins H. ‘The cure for us is a lot of things’: how young people with prader-willi syndrome view themselves and future clinical trials. J Appl Res Intellectual Disabil (2022) 35(2):460–70. doi: 10.1111/jar.12950 34904341

[14] Wong SB Wang TS Tsai WH Tzeng IS Tsai LP. Parenting stress in families of children with prader–willi syndrome. Am J Med Genet Part A (2021) 185(1):83–9. doi: 10.1002/ajmg.a.61915 33043996

[15] Holland AJ Aman LC Whittington JE. Defining mental and behavioural disorders in genetically determined neurodevelopmental syndromes with particular reference to prader-willi syndrome. Genes (2019) 10(12):1025. doi: 10.3390/genes10121025 31835392 PMC6947448

[16] Rice LJ Einfeld SL Hu N Carter CS. A review of clinical trials of oxytocin in prader–willi syndrome. Curr Opin Psychiatry (2018) 31(2):123–7. doi: 10.1097/YCO.0000000000000391 29206687

[17] Swaab D Purba J Hofman M. Alterations in the hypothalamic paraventricular nucleus and its oxytocin neurons (Putative satiety cells) in prader-willi syndrome: a study of five cases. J Clin Endocrinol Metab (1995) 80(2):573–9. doi: 10.1210/jc.80.2.573 7852523

[18] Carter CS. Oxytocin pathways and the evolution of human behavior. Annu Rev Psychol (2014) 65:17–39. doi: 10.1146/annurev-psych-010213-115110 24050183

[19] Carter CS Kenkel WM MacLean EL Wilson SR Perkeybile AM Yee JR. Is oxytocin “Nature’s medicine”? Pharmacol Rev (2020) 72(4):829–61. doi: 10.1124/pr.120.019398 PMC7495339 32912963

[20] Donaldson ZR Young LJ. Oxytocin, vasopressin, and the neurogenetics of sociality. Science (2008) 322(5903):900–4. doi: 10.1126/science.1158668 18988842

[21] De Vries G Buijs R Van Leeuwen F. Sex differences in vasopressin and other neurotransmitter systems in the brain. Prog Brain Res (1984) 61:185–203. doi: 10.1016/S0079-6123(08)64435-0 6152059

[22] Grinevich V Ludwig M. The multiple faces of the oxytocin and vasopressin systems in the brain. J Neuroendocrinol (2021) 33(11):e13004. doi: 10.1111/jne.13004 34218479

[23] Baribeau DA Anagnostou E. Oxytocin and vasopressin: linking pituitary neuropeptides and their receptors to social neurocircuits. Front Neurosci (2015) 9:335. doi: 10.3389/fnins.2015.00335 26441508 PMC4585313

[24] Griffante C Green A Curcuruto O Haslam CP Dickinson BA Arban R. Selectivity of d [Cha4] avp and Ssr149415 at human vasopressin and oxytocin receptors: evidence that Ssr149415 is a mixed vasopressin V1b/Oxytocin receptor antagonist. Br J Pharmacol (2005) 146(5):744–51. doi: 10.1038/sj.bjp.0706383 PMC1751202 16158071

[25] Landgraf R Neumann ID. Vasopressin and oxytocin release within the brain: a dynamic concept of multiple and variable modes of neuropeptide communication. Front Neuroendocrinol (2004) 25(3-4):150–76. doi: 10.1016/j.yfrne.2004.05.001 15589267

[26] Bittel DC Kibiryeva N Sell SM Strong TV Butler MG. Whole genome microarray analysis of gene expression in prader–willi syndrome. Am J Med Genet Part A (2007) 143(5):430–42. doi: 10.1002/ajmg.a.31606 PMC5467864 17236194

[27] Salles J Eddiry S Lacassagne E Laurier V Molinas C Bieth É. Patients with pws and related syndromes display differentially methylated regions involved in neurodevelopmental and nutritional trajectory. Clin Epigenet (2021) 13(1):1–12. doi: 10.1186/s13148-021-01143-0 PMC8361855 34389046

[28] Heseding HM Jahn K Eberlein CK Wieting J Maier HB Proskynitopoulos PJ. Distinct promoter regions of the oxytocin receptor gene are hypomethylated in prader-willi syndrome and in prader-willi syndrome associated psychosis. Trans Psychiatry (2022) 12(1):1–10. doi: 10.1038/s41398-022-02014-9 PMC9187685 35688807

[29] Camfferman D McEvoy RD O’Donoghue F Lushington K. Prader willi syndrome and excessive daytime sleepiness. Sleep Med Rev (2008) 12(1):65–75. doi: 10.1016/j.smrv.2007.08.005 18201664

[30] Cassidy SB Driscoll DJ. Prader–willi syndrome. Eur J Hum Genet (2009) 17(1):3–13. doi: 10.1038/ejhg.2008.165 18781185 PMC2985966

[31] Bochukova EG Lawler K Croizier S Keogh JM Patel N Strohbehn G. A transcriptomic signature of the hypothalamic response to fasting and bdnf deficiency in prader-willi syndrome. Cell Rep (2018) 22(13):3401–8. doi: 10.1016/j.celrep.2018.03.018 PMC5896230 29590610

[32] Oztan O Zyga O Stafford DE Parker KJ. Linking oxytocin and arginine vasopressin signaling abnormalities to social behavior impairments in prader-willi syndrome. Neurosci Biobehav Rev (2022) 142:104870. doi: 10.1016/j.neubiorev.2022.104870 36113782 PMC11024898

[33] Höybye C. Endocrine and metabolic aspects of adult prader–willi syndrome with special emphasis on the effect of growth hormone treatment. Growth hormone IGF Res (2004) 14(1):1–15. doi: 10.1016/j.ghir.2003.09.003 14700552

[34] Höybye C Barkeling B Espelund U Petersson M Thorén M. Peptides associated with hyperphagia in adults with prader–willi syndrome before and during gh treatment. Growth Hormone IGF Res (2003) 13(6):322–7. doi: 10.1016/S1096-6374(03)00077-7 14624765

[35] Johnson L Manzardo AM Miller JL Driscoll DJ Butler MG. Elevated plasma oxytocin levels in children with prader–willi syndrome compared with healthy unrelated siblings. Am J Med Genet Part A (2016) 170(3):594–601. doi: 10.1002/ajmg.a.37488 26615966 PMC6679917

[36] Martin A State M Anderson GM Kaye WM Hanchett JM McConaha CW. Cerebrospinal fluid levels of oxytocin in prader–willi syndrome: a preliminary report. Biol Psychiatry (1998) 44(12):1349–52. doi: 10.1016/S0006-3223(98)00190-5 9861478

[37] Damen L Grootjen LN Juriaans AF Donze SH Huisman TM Visser JA. Oxytocin in young children with prader-willi syndrome: results of a randomized, double-blind, placebo-controlled, crossover trial investigating 3 months of oxytocin. Clin Endocrinol (2021) 94(5):774–85. doi: 10.1111/cen.14387 PMC8246775 33296519

[38] Kuppens R Donze S Hokken-Koelega A. Promising effects of oxytocin on social and food-related behaviour in young children with prader–willi syndrome: a randomized, double-blind, controlled crossover trial. Clin Endocrinol (2016) 85(6):979–87. doi: 10.1111/cen.13169 27486141

[39] Miller JL Tamura R Butler MG Kimonis V Sulsona C Gold JA. Oxytocin treatment in children with prader–willi syndrome: a double-blind, placebo-controlled, crossover study. Am J Med Genet Part A (2017) 173(5):1243–50. doi: 10.1002/ajmg.a.38160 PMC5828021 28371242

[40] Tauber M Mantoulan C Copet P Jauregui J Demeer G Diene G. Oxytocin may be useful to increase trust in others and decrease disruptive behaviours in patients with prader-willi syndrome: a randomised placebo-controlled trial in 24 patients. Orphanet J Rare Dis (2011) 6(1):1–6. doi: 10.1186/1750-1172-6-47 21702900 PMC3141367

[41] Tauber M Boulanouar K Diene G Çabal-Berthoumieu S Ehlinger V Fichaux-Bourin P. The use of oxytocin to improve feeding and social skills in infants with prader–willi syndrome. Pediatrics (2017) 139(2):e20162976. doi: 10.1542/peds.2016-2976 28100688

[42] Einfeld SL Smith E McGregor IS Steinbeck K Taffe J Rice LJ. A double-blind randomized controlled trial of oxytocin nasal spray in prader willi syndrome. Am J Med Genet Part A (2014) 164(9):2232–9. doi: 10.1002/ajmg.a.36653 24980612

[43] Hollander E Levine KG Ferretti CJ Freeman K Doernberg E Desilva N. Intranasal oxytocin versus placebo for hyperphagia and repetitive behaviors in children with prader-willi syndrome: a randomized controlled pilot trial. J Psychiatr Res (2021) 137:643–51. doi: 10.1016/j.jpsychires.2020.11.006 33190843

[44] Rosell DR Siever LJ. The neurobiology of aggression and violence. CNS spectrums (2015) 20(3):254–79. doi: 10.1017/S109285291500019X 25936249

[45] Carter CS Grippo AJ Pournajafi-Nazarloo H Ruscio MG Porges SW. Oxytocin, vasopressin and sociality. Prog Brain Res (2008) 170:331–6. doi: 10.1016/S0079-6123(08)00427-5 18655893

[46] Gabreeüls BTF Swaab D De Kleijn D Seidah N Van de Loo J-W Van de Ven W. Attenuation of the polypeptide 7b2, prohormone convertase Pc2, and vasopressin in the hypothalamus of some prader-willi patients: indications for a processing defect. J Clin Endocrinol Metab (1998) 83(2):591–9. doi: 10.1210/jc.83.2.591 9467579

[47] Yamada K Watanabe M Suzuki K. Reduced pituitary volume with relative T1 shortening correlates with behavior in prader-willi syndrome. Biomarkers Neuropsychiatry (2021) 5:100039. doi: 10.1016/j.bionps.2021.100039

[48] Wechsler D. Wechsler abbreviated scale of intelligence. (London, England: Harcourt Assessment). (1999).

[49] Einfeld S Tonge BJ. Developmental behaviour checklist (Dbc). Victoria (2002).

[50] Rice LJ Lagopoulos J Brammer M Einfeld SL. Reduced gamma-aminobutyric acid is associated with emotional and behavioral problems in prader–willi syndrome. Am J Med Genet Part B: Neuropsychiatr Genet (2016) 171(8):1041–8. doi: 10.1002/ajmg.b.32472 27338833

[51] Clarke AR Tonge BJ Einfeld SL Mackinnon A. Assessment of change with the developmental behaviour checklist. J Intellectual Disability Res (2003) 47(3):210–2. doi: 10.1046/j.1365-2788.2003.00470.x 12603518

[52] Fehnel S Brown TM Nelson L Chen A Kim D Roof E. Development of the hyperphagia questionnaire for use in prader-willi syndrome clinical trials. Value Health (2015) 18(3):A25. doi: 10.1016/j.jval.2015.03.154

[53] Dykens EM Maxwell MA Pantino E Kossler R Roof E. Assessment of hyperphagia in prader-willi syndrome. Obesity (2007) 15(7):1816–26. doi: 10.1038/oby.2007.216 17636101

[54] Ekman P Friesen WV. Unmasking the face: a guide to recognizing emotions from facial clues. (Cambridge, MA: Malor Books). (1975).

[55] Whittington J Holland T. Recognition of emotion in facial expression by people with prader–willi syndrome. J Intellectual Disability Res (2011) 55(1):75–84. doi: 10.1111/j.1365-2788.2010.01348.x 21121995

[56] Butler J Whittington J Holland A Boer H Clarke D Webb T. Prevalence of, and risk factors for, physical ill-health in people with prader-willi syndrome: a population-based study. Dev Med Child Neurol (2002) 44(4):248–55. doi: 10.1017/S001216220100202X 11995893

[57] Williams K Scheimann A Sutton V Hayslett E Glaze DG. Sleepiness and sleep disordered breathing in prader-willi syndrome: relationship to genotype, growth hormone therapy, and body composition. J Clin Sleep Med (2008) 4(2):111–8. doi: 10.5664/jcsm.27126 PMC2335405 18468308

[58] Patel VP Patroneva A Glaze DG Davis MSK Merikle E Revana A. Establishing the content validity of the epworth sleepiness scale for children and adolescents in prader-willi syndrome. J Clin Sleep Med (2022) 18(2):485–96. doi: 10.5664/jcsm.9632 PMC8804999 34437052

[59] Carter CS. Sex differences in oxytocin and vasopressin: implications for autism spectrum disorders? Behav Brain Res (2007) 176(1):170–86. doi: 10.1016/j.bbr.2006.08.025 17000015

[60] MacLean EL Wilson SR Martin WL Davis JM Nazarloo HP Carter CS. Challenges for measuring oxytocin: the blind men and the elephant? Psychoneuroendocrinology (2019) 107:225–31. doi: 10.1016/j.psyneuen.2019.05.018 PMC6634994 31163380

[61] Plasencia G Luedicke JM Nazarloo HP Carter CS Ebner NC. Plasma oxytocin and vasopressin levels in young and older men and women: functional relationships with attachment and cognition. Psychoneuroendocrinology (2019) 110:104419. doi: 10.1016/j.psyneuen.2019.104419 31606581 PMC6943921

[62] Engel S Klusmann H Ditzen B Knaevelsrud C Schumacher S. Menstrual cycle-related fluctuations in oxytocin concentrations: a systematic review and meta-analysis. Front Neuroendocrinol (2019) 52:144–55. doi: 10.1016/j.yfrne.2018.11.002 30458185

[63] Tabak BA Leng G Szeto A Parker KJ Verbalis JG Ziegler TE. Advances in human oxytocin measurement: challenges and proposed solutions. Mol Psychiatry (2022) 28(1):127–40. doi: 10.1038/s41380-022-01719-z PMC9812775 35999276

[64] Sinnema M Maaskant MA van Schrojenstein Lantman-de Valk HM van Nieuwpoort IC Drent ML Curfs LM. Physical health problems in adults with prader–willi syndrome. Am J Med Genet Part A (2011) 155(9):2112–24. doi: 10.1002/ajmg.a.34171 21834028

[65] Al-Ayadhi LY. Altered oxytocin and vasopressin levels in autistic children in central Saudi Arabia. Neurosci J (2005) 10(1):47–50. 22473184

[66] Boso M Emanuele E Politi P Pace A Arra M di Nemi SU. Reduced plasma apelin levels in patients with autistic spectrum disorder. Arch Med Res (2007) 38(1):70–4. doi: 10.1016/j.arcmed.2006.08.003 17174726

[67] Carson DS Garner JP Hyde SA Libove RA Berquist SW Hornbeak KB. Arginine vasopressin is a blood-based biomarker of social functioning in children with autism. PloS One (2015) 10(7):e0132224. doi: 10.1371/journal.pone.0132224 26200852 PMC4511760

[68] Miller M Bales KL Taylor SL Yoon J Hostetler CM Carter CS. Oxytocin and vasopressin in children and adolescents with autism spectrum disorders: sex differences and associations with symptoms. Autism Res (2013) 6(2):91–102. doi: 10.1002/aur.1270 23413037 PMC3657571

[69] Zhang H-F Dai Y-C Wu J Jia M-X Zhang J-S Shou X-J. Plasma oxytocin and arginine-vasopressin levels in children with autism spectrum disorder in China: associations with symptoms. Neurosci Bull (2016) 32(5):423–32. doi: 10.1007/s12264-016-0046-5 PMC5563759 27342432

[70] Wilczyński KM Zasada I Siwiec A Janas-Kozik M. Differences in oxytocin and vasopressin levels in individuals suffering from the autism spectrum disorders vs general population–a systematic review. Neuropsychiatr Dis Treat (2019) 15:2613–20. doi: 10.2147/NDT.S207580 PMC6750159 31571878

[71] Parker KJ Oztan O Libove RA Mohsin N Karhson DS Sumiyoshi RD. A randomized placebo-controlled pilot trial shows that intranasal vasopressin improves social deficits in children with autism. Sci Trans Med (2019) 11(491):eaau7356. doi: 10.1016/j.neubiorev.2022.104870 PMC6716148 31043522

[72] Hollander E Jacob S Jou R McNamara N Sikich L Tobe R. Balovaptan vs placebo for social communication in childhood autism spectrum disorder: a randomized clinical trial. JAMA Psychiatry (2022) 79(8):760–9. doi: 10.1001/jamapsychiatry.2022.1717 PMC9260643 35793101

[73] Borie AM Dromard Y Guillon G Olma A Manning M Muscatelli F. Correction of vasopressin deficit in the lateral septum ameliorates social deficits of mouse autism model. J Clin Invest (2021) 131(2):e144450. doi: 10.1172/JCI144450 33232306 PMC7810497

[74] John S Jaeggi AV. Oxytocin levels tend to be lower in autistic children: a meta-analysis of 31 studies. Autism (2021) 25(8):2152–61. doi: 10.1177/13623613211034375 34308675

[75] Moerkerke M Peeters M de Vries L Daniels N Steyaert J Alaerts K. Endogenous oxytocin levels in autism–a meta-analysis. Brain Sci (2021) 11(11):1545. doi: 10.3390/brainsci11111545 34827545 PMC8615844

[76] Valstad M Alvares GA Egknud M Matziorinis AM Andreassen OA Westlye LT. The correlation between central and peripheral oxytocin concentrations: a systematic review and meta-analysis. Neurosci Biobehav Rev (2017) 78:117–24. doi: 10.1016/j.neubiorev.2017.04.017 28442403

[77] Chen Q Zhuang J Zuo R Zheng H Dang J Wang Z. Exploring associations between postpartum depression and oxytocin levels in cerebrospinal fluid, plasma and saliva. J Affect Disord (2022) 315:198–205. doi: 10.1016/j.jad.2022.07.052 35917937

[78] Martin J Kagerbauer SM Gempt J Podtschaske A Hapfelmeier A Schneider G. Oxytocin levels in saliva correlate better than plasma levels with concentrations in the cerebrospinal fluid of patients in neurocritical care. J Neuroendocrinol (2018) 30(5):e12596. doi: 10.1111/jne.12596 29611254

[79] Dykens EM Roof E Hunt-Hawkins H Dankner N Lee EB Shivers CM. Diagnoses and characteristics of autism spectrum disorders in children with prader-willi syndrome. J Neurodev Disord (2017) 9(1):1–12. doi: 10.1186/s11689-017-9200-2 28592997 PMC5458479

[80] Engel S Laufer S Miller R Niemeyer H Knaevelsrud C Schumacher S. Demographic, sampling-and assay-related confounders of endogenous oxytocin concentrations: a systematic review and meta-analysis. Front Neuroendocrinol (2019) 54:100775. doi: 10.1016/j.yfrne.2019.100775 31351080

[81] Marazziti D Carter CS Carmassi C Della Vecchia A Mucci F Pagni G. Sex matters: the impact of oxytocin on healthy conditions and psychiatric disorders. Compr Psychoneuroendocrinol (2023) 13:100165. doi: 10.1016/j.cpnec.2022.100165 36590869 PMC9800179

[82] McCullough ME Churchland PS Mendez AJ. Problems with measuring peripheral oxytocin: can the data on oxytocin and human behavior be trusted? Neurosci Biobehav Rev (2013) 37(8):1485–92. doi: 10.1016/j.neubiorev.2013.04.018 23665533

[83] Carter CS Dantzer R. Love and fear: a special issue. Compr Psychoneuroendocrinol. (2022). p. 100151. 10.1016/j.cpnec.2022.100151 PMC9363639 35967926

[84] Brandtzaeg OK Johnsen E Roberg-Larsen H Seip KF MacLean EL Gesquiere LR. Proteomics tools reveal startlingly high amounts of oxytocin in plasma and serum. Sci Rep (2016) 6(1):31693. doi: 10.1038/srep31693 27528413 PMC4985690

[85] Chu C Hammock EA Joiner TE. Unextracted plasma oxytocin levels decrease following in-laboratory social exclusion in young adults with a suicide attempt history. J Psychiatr Res (2020) 121:173–81. doi: 10.1016/j.jpsychires.2019.11.015 PMC6939138 31835187

[86] Saxbe D Khaled M Horton KT Mendez A. Maternal prenatal plasma oxytocin is positively associated with prenatal psychological symptoms, but method of immunoassay extraction may affect results. Biol Psychol (2019) 147:107718. doi: 10.1016/j.biopsycho.2019.107718 31199947

