# Supplementary material for: The relationship between endogenous oxytocin and vasopressin levels and the Prader-Willi syndrome behaviour phenotype
Source: Front Endocrinol (Lausanne). 2023 May 29;14:1183525. doi: 10.3389/fendo.2023.1183525 (PMC10259653; doi:10.3389/fendo.2023.1183525)
Supplement: Supplementary file 1 [file Table_1.docx]

**Supplementary Table 1:** Non-transformed table of descriptive statistics for the full cohort

|  | N | Range | Minimum | Maximum | Mean | Std. Deviation |
| --- | --- | --- | --- | --- | --- | --- |
| Plasma OT | 60 | 716.80 | 34.00 | 750.80 | 198.5427 | 109.60731 |
| Plasma AVP | 60 | 253.26 | 17.74 | 271.00 | 70.7200 | 57.12412 |
| Saliva OT | 56 | 39.25 | 7.69 | 46.94 | 23.8060 | 7.44059 |
| Time of blood draw | 60 | 9.00 | 9.00 | 18.00 | 13.2500 | 1.99682 |

**Supplementary Table 2**: Non-transformed table of descriptive statistics by group

| PWS or Control | | N | Range | Minimum | Maximum | Mean | Std. Deviation |
| --- | --- | --- | --- | --- | --- | --- | --- |
| PWS | Plasma OT | 30 | 327.00 | 34.00 | 361.00 | 187.4677 | 76.32890 |
|  | Plasma AVP | 30 | 244.86 | 17.74 | 262.60 | 56.1243 | 48.45586 |
|  | Saliva OT | 26 | 25.20 | 15.97 | 41.17 | 23.4600 | 6.58121 |
|  | Time of blood draw | 30 | 8.00 | 9.00 | 17.00 | 13.2667 | 1.85571 |
| Control | Plasma OT | 30 | 667.65 | 83.15 | 750.80 | 209.6177 | 135.50617 |
|  | Plasma AVP | 30 | 240.44 | 30.56 | 271.00 | 85.3157 | 62.04937 |
|  | Saliva OT | 30 | 39.25 | 7.69 | 46.94 | 24.1059 | 8.21335 |
|  | Time of blood draw | 30 | 7.00 | 11.00 | 18.00 | 13.2333 | 2.16051 |

**Supplementary Table 3:** DBC-A subscales and corresponding example items

| **Subscale** | **Example Items** |
| --- | --- |
| Disruptive | Has temper tantrums, eg stamps feet, slams doors. *  Kicks, hits or injures others.  Mood changes rapidly for no apparent reason.  Stubborn, disobedient or unco-operative. *  Cries easily for no reason, or over small upsets. *  Upset and distressed over small changes in routine and environment. *  Tense, anxious, worried. |
| Communication and anxiety disturbance | Repeats the same word or phrase over and over.  Bizarre speech.  Gets obsessed with an idea or activity. *  Upset and distressed over small changes in routine or environment. * |
| Self-absorbed | Soils outside toilet though toilet trained. Smears or plays with faeces.  Laughs or giggles for no obvious reason.  Eats non-food items, eg dirt, grass, soap. *  Gorges food. Will do anything to get food, eg takes food out of garbage bins or steals food. *  Smells, tastes or licks objects. * |
| Antisocial | Steals. *  Tells lies. *  Impulsive, acts before thinking.  Stubborn, disobedient or unco-operative. *  Very bossy.  Abusive. Swears at others.  Gorges food. Will do anything to get food, eg takes food out of garbage bins or steals food. * |
| Depressive | Sleeps too much or overly drowsy. *  Moves slowly, underactive, does little, eg only sits and watches others. *  Appears depressed, downcast or unhappy.  Has become confused or forgetful. |
| Social relating | Prefers to do things on his/her own. Tends to be a loner.  Shy. *  Arranges objects or routine in a strict order. |
| Unallocated DBC-A items | Scratches or picks her/his skin. *  Underreacts to pain. * |

* Items that occur more commonly in PWS than the general intellectual disability population.

**
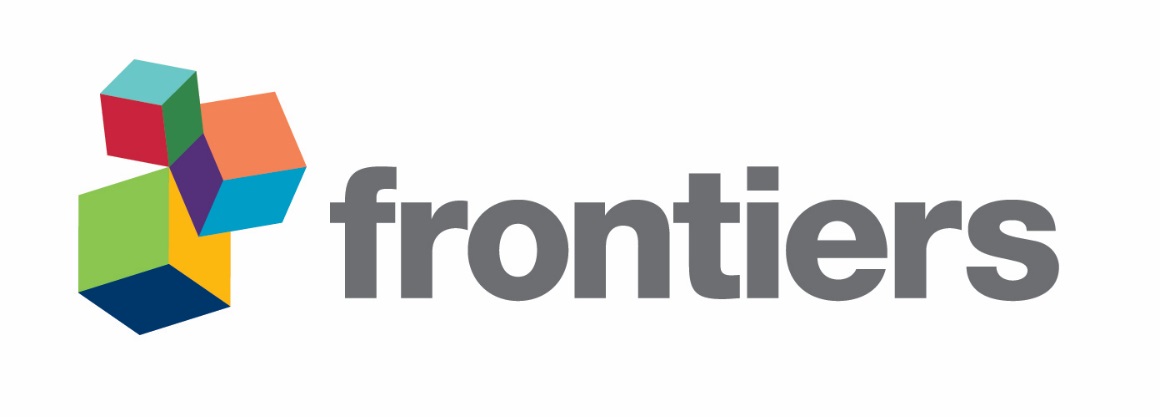
**
